# Utilization and out-of-pocket expenses of primary care among the multimorbid elderly in China: A two-part model with nationally representative data

**DOI:** 10.3389/fpubh.2022.1057595

**Published:** 2022-11-24

**Authors:** Yuehua Chen, Wenbin Liu

**Affiliations:** Department of Health Management, School of Health Management, Fujian Medical University, Fuzhou, China

**Keywords:** multimorbidity, primary care, out-of-pocket expenses, two-part model, grossman theory, CHARLS, the elderly

## Abstract

**Background:**

Multimorbidity has become an essential public health issue that threatens human health and leads to an increased disease burden. Primary care is the prevention and management of multimorbidity by providing continuous, comprehensive patient-centered services. Therefore, the study aimed to investigate the determinants of primary care utilization and out-of-pocket expenses (OOPE) among multimorbid elderly to promote rational utilization of primary care and reduce avoidable economic burdens.

**Methods:**

The study used data from CHARLS 2015 and 2018, which included a total of 4,384 multimorbid elderly aged 60 and above. Guided by Grossman theory, determinants such as education, gender, marriage, household economy, and so on were included in this study. A two-part model was applied to evaluate primary care utilization and OOPE intensity in multimorbid populations. And the robustness testing was performed to verify research results.

**Results:**

Primary care visits rate and OOPE indicated a decline from 2015 to 2018. Concerning primary outpatient care, the elderly who were female (*OR* = 1.51*, P* < 0.001), married (*OR* = 1.24, *P* < 0.05), living in rural areas (*OR* = 1.77, *P* < 0.001) and with poor self-rated health (*OR* = 2.23, *P* < 0.001) had a significantly higher probability of outpatient utilization, whereas those with middle school education (*OR* = 0.61, *P* < 0.001) and better household economy (*OR* = 0.96, *P* < 0.001) had a significantly less likelihood of using outpatient care. Rural patients (β = −0.72, *P* < 0.05) may have lower OOPE, while those with better household economy (β = 0.29, *P* < 0.05; β = 0.58, *P* < 0.05) and poor self-rated health (β = 0.62, *P* < 0.001) occurred higher OOPE. Regarding primary inpatient care, adults who were living in rural areas (*OR* = 1.48, *P* < 0.001), covered by Urban Employee Basic Medical Insurance (UEBMI) or Urban Rural Basic Medical Insurance (URBMI) (*OR* = 2.46, *P* < 0.001; *OR* = 1.81, *P* < 0.001) and with poor self-rated health (*OR* = 2.30, *P* < 0.001) had a significantly higher probability of using inpatient care, whereas individuals who were female (*OR* = 0.74, *P* < 0.001), with middle school education (*OR* = 0.40, *P* < 0.001) and better household economy (*OR* = 0.04, *P* < 0.001) had a significantly lower tendency to use inpatient care. Significantly, more OOPE occurred by individuals who were women (β = 0.18, *P* < 0.05) and with better household economy (β = 0.40, *P* < 0.001; β = 0.62, *P* < 0.001), whereas those who were covered by URBMI (β = −0.25, *P* < 0.05) and satisfied with their health (β = −0.21, *P* < 0.05) had less OOPE.

**Conclusion:**

To prompt primary care visits and reduce economic burden among subgroups, more policy support is in need, such as tilting professional medical staff and funding to rural areas, enhancing awareness of disease prevention among vulnerable groups and so on.

## Introduction

With changing lifestyles, increasing personal risk factors, and aging populations, multimorbidity has replaced infectious diseases as the major health burden in older adults, posing a clear challenge to the public health systems in all countries (1). Multimorbidity is defined as the coexistence of two or more chronic diseases in a person (2, 3), with global prevalence ranging from 12.9% (the whole population) to 95.1% (those aged 65 years and older) (4), while the prevalence in low-and-middle-income countries (LMICs) is on the rise (5–7). Multimorbidity is closely related to disability, decreased quality of life, premature death, unplanned hospitalizations, and increased consumption of health resources, which imposes a heavy strain on individuals and healthcare systems (7, 8).

To address serious conditions caused by multimorbidity, primary care has been strongly recommended as it placed an emphasis on patient-centered comprehensive, integrated, and continuous care services, which will be a critical link in the prevention and management of chronic disease (9, 10), and contribute to fulfill a large number of individual healthcare demands and reduce potential economic catastrophe led by multimorbidity. Currently, many countries are in the process of reforming the primary healthcare system. The National Health Service (NHS) in the UK is one of the most mature systems in the world, characterized by a rigorous community-based first visit and general practitioner based on referrals system (11). In Japan, the government is trying to establish a community-based integrated care system to provide supportive treatment, as the boundaries between primary care and secondary and tertiary care are blurred (10). In 2015, China implemented a hierarchical medical system centered on primary diagnosis and two-way referral to efficiently utilize medical resources. However, the system has encountered challenges. Residents are influenced by their own socioeconomic status or the service capacity of medical facilities to bypass primary care institutions for treatment at higher-level institutions (12, 13), causing lower visits rate in primary care institutions. The national bed occupancy rate in primary care institutions is just 54.7% (14). In Shanghai, only 53.48% of the urban population went to primary medical institutions for treatment (15), far lower than the 90% visits rate in the UK. Under this context, it is in urgent need to reveal the potential obstacles and economic burdens of primary care utilization to alter the attitudes of older adults toward primary medical institutions and revitalize medical resources.

Previous studies have scattered noted and made some progress the factors of healthcare utilization and expenses in different groups, which are age, gender, education, income, self-rated health, health insurance, etc. On the one hand, age was considered to be a key factor affecting the willingness to seek medical treatment for multimorbid patients (3, 16), while related studies showed no relationship between healthcare utilization and age (17). Women (16, 18) and those covered by health insurance (19) were generally more likely to use healthcare. The impact of education, income and occupation on healthcare utilization varies widely across countries. Studies in high-income countries (HICs) investigated that such factors were not related to primary care visits (20), while education and income were positively correlated with primary care visits in LMICs (21, 22). On the other hand, although age has also been pointed out as a favorable factor affecting costs (23, 24), other studies opposed the argument (25, 26). Patients who are married and living in underdeveloped areas had higher medical expenses. Self-rated health and socioeconomic factors such as income and education played a vital role in medical expenditure (19). The heterogeneity exhibited by the findings reflected the degrees of multimorbidity and the differences in healthcare systems across countries. To date, although previous research has focused on the determinants of healthcare utilization and expenditures in different groups, little detailed attention has been paid to the utilization of primary outpatient and inpatient services among the multimorbid elderly (5, 27). Additionally, research methods are also homogeneous, with most analyses of medical costs using multiple linear regression (28, 29) and quantile regression (7, 30), which takes less account of the characteristics, namely the presence of a small number of zeros and non-normal distribution, resulting in biased estimates (31). Finally, the inclusion of factors on health care utilization and expenditure is fragmented and lacks a comprehensive approach for overall consideration in previous studies. Furthermore, ignoring the impact of individual health demands, as an antecedent demand (32), on utilization and expenditure behavior may give rise to difficulties in screening the true and reasonable use and costs.

Therefore, to fill the research gaps as mentioned above, the study will focus on the primary care utilization and expenses among older adults with multimorbidity using a two-part model, as well as validate determinants on utilization and expenditures based on the Grossman theory. The outcome of this study will benefit to understand the barriers and financial burden of primary care utilization among multimorbid groups, and provide targeted recommendations to further promote appropriate utilization and avoid excessive medical expenditures, ultimately contributing to rational allocation and efficient use of primary medical resources.

## Materials and methods

### Theory

To overcome the shortcoming of ignoring the impact of health demands on utilization and expenditure of health services, it is more appropriate to investigate medical use and expenditures in multimorbid groups from the perspective of health demand. Thus, Grossman theory, which affirms that healthcare utilization and spending behavior stems from the individual demand, was applied in this study to provide theoretical guidance to describe the factors influencing healthcare utilization choices and expenditures among multimorbid patients. This theory asserts that health can be viewed as a durable stock of capital that declines with age (32). In order to obtain or maintain health, individuals restore health and improve work productivity through using healthcare services and managing risk factor.

Due to the multi-layered and complex of medical services, Grossman has hypothesized that health demand is influenced by factors such as age, education, gender, marital status, income, and personal behavior (such as smoking, diet, and exercise) and so on (33, 34). The main assumptions have also been confirmed by several empirical studies. (i) The increase in age leads to a decline in personal health, which drives more medical demands. A study using Grossman-PLS model to predict key factors on the growth of healthcare spending in the middle east region revealed that aging and the relative wage rate were the statistically significant indicators (35). (ii) As individuals' incomes increase, their health and medical demands increase. Hartwig et al. (36) used macro panel data to test the validity of Grossman theory and confirmed that real wages were considered to be a robust predictor. (iii) More educated individuals have less demand for medical services and lower health expenditures. An analysis of health service demand in Costa Rica based on Grossman theory found that the key determinants of health care utilization were education level and self-assessed health (37). A study of health equality in social groups through the lens of health capital theory emphasized the deterministic role of education on health (38). (iv) The intervention of health insurance drives the patients' medical demands. On the basis of the health demand model, Sorkin argued that health insurance not only played a certain part in reducing the health care price, but also stimulated individual demand for medical services (39).

### Data

The study used data from two waves of the China Health and Retirement Longitudinal Study (CHARLS) in 2015 and 2018. CHARLS is a biennial follow-up survey conducted by the National Development Research Institute at the Peking University, targeting Chinese residents aged 45 or older and their households. It is a comprehensive and informative database that covers many aspects of socioeconomic status and personal health status through one-to-one interviews with structured questionnaires in China. The baseline survey was conducted in 2011 using the probability proportional to size (PPS) sampling to select respondents from 150 counties, 450 villages, and about 17,000 individuals across 28 provinces in China. More detailed information can be found on the official CHARLS website (https://charls.charlsdata.com/index/zh-cn.html).

**Figure 1:**
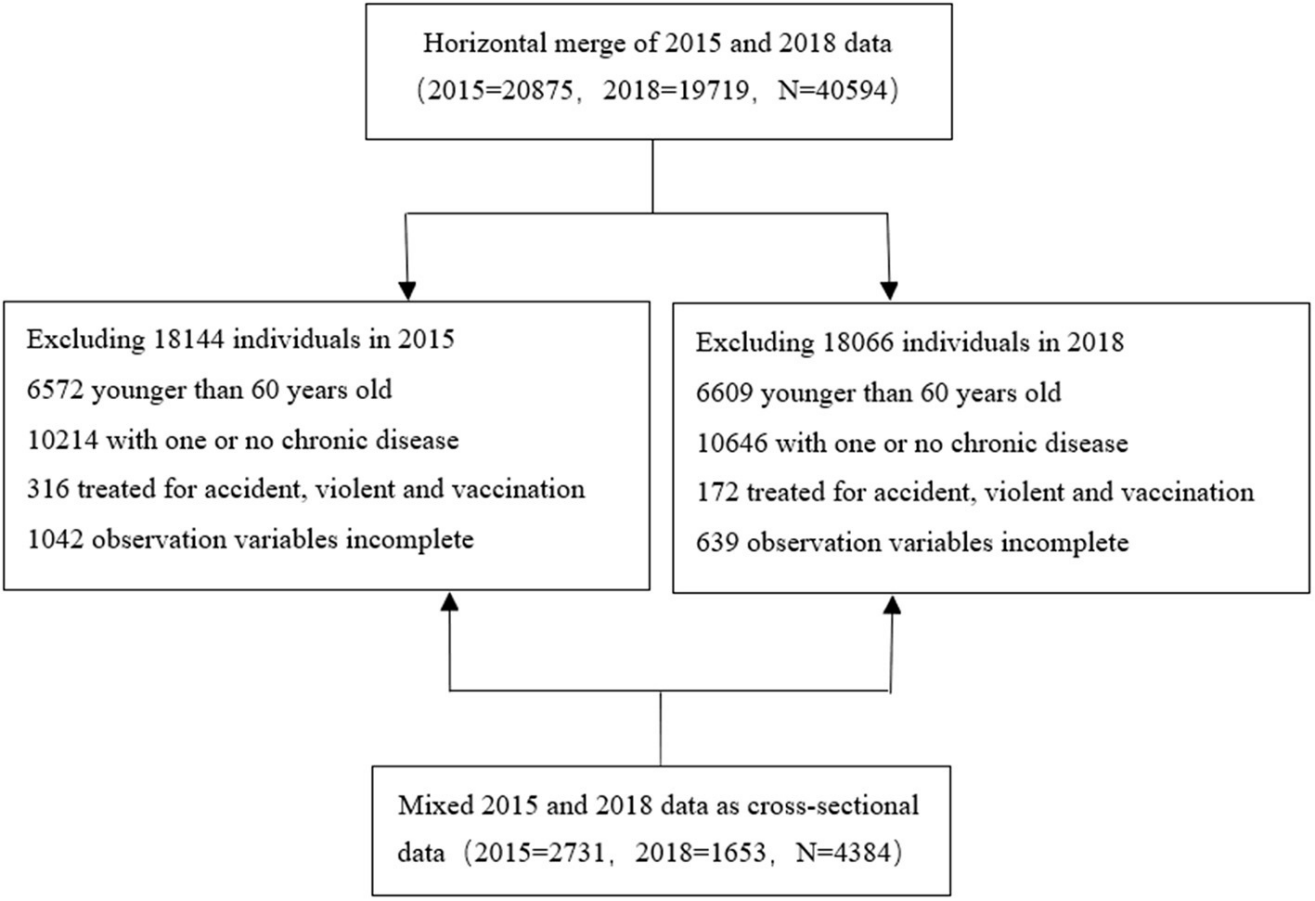
Sample inclusion flow chart.

According to the study design, samples from 2015 and 2018 were excluded if they met any of the following criteria. (i) Individuals aged <60 years old. (ii) Individuals with one or no chronic disease (more details were shown in 2.3.1 below). (iii) Individuals seeking treatment due to violence, accident, vaccination, etc. (iv) Individuals with incomplete sample information. The final data set included 4,384 respondents in 2 waves, with 2,731 individuals in 2015 and 1,653 individuals in 2018, to form a pooled cross-sectional data. The details of the process are shown in Figure 1.

### Measurements

#### Chronic diseases and multimorbidity

A total of 14 chronic diseases were contained in the CHARLS: hypertension, dyslipidemia [elevation of low-density lipoprotein, triglycerides (TGs), and total cholesterol, or a low high-density lipoprotein level], diabetes or high blood sugar, cancer or malignant tumor (excluding minor skin cancers), chronic lung diseases such as chronic bronchitis, emphysema (excluding tumors, or cancer), liver disease (except fatty liver, tumors, and cancer), heart attack (such as coronary heart disease, angina, congestive heart failure, or other heart problems), stroke, kidney disease (except for tumor or cancer), stomach or other digestive disease (except for tumor or cancer), emotional or psychiatric problems, memory-related disease, arthritis or rheumatism, asthma. The question in CHARLS regarding the presence of chronic diseases is “Have you ever been diagnosed with these chronic diseases by a doctor?.” If respondents answered two or more chronic diseases, they were judged to be multimorbid.

#### Dependent variables

The outcome variables included in this study are primary outpatient care utilization and OOPE, primary inpatient care utilization and OOPE. The primary outpatient care utilization was measured by “Which medical provider did you visit most recently for outpatient care during the past month?” The primary inpatient care utilization was evaluated by “Which type of health facilities did you most recent visit for last inpatient care (hospital admissions) in the past year?” If they visited a community healthcare center, township hospital, health care post, or village clinic/private clinic, respondents were defined as having primary outpatient or inpatient care utilization and continued to ask about the OOPE.

The OOPE of primary outpatient and inpatient, defined as the expenditure after being reimbursed by health insurance, was measured by “How much did you pay out of pocket, after reimbursement from insurance for last outpatient care in the past month?” and “How much did you pay out of pocket for your last hospitalization in the past year?” in CHARLS. The first part of primary care utilization is a dichotomous variable (used/not used), and the second part of medical expenditure is a continuous variable (≥0). Those who did not use primary care were excluded from the second part.

#### Independent variables

Based on Grossman theory, this research selected ten variables from CHARLS: gender, age (60~69, 70~79, 80~), education (illiterate, primary school, middle school, high school and above), marital status, residence, household economy (monthly per capita household consumption expenditure), health insurance [uninsured, UEBMI (Urban Employee Basic Medical Insurance), URBMI (Urban Rural Basic Medical Insurance), more than one, other insurance.], self-rated health (very good, good, fair, and poor), health satisfaction, and exercise. This study adopted household economy, which was considered more reliable, rather than personal income. Previous studies have shown that most rural older adults have unstable or almost no income, whereas the household economy can faithfully reflect the financial support that patients' families can provide during treatment (40, 41). Besides, there was a lack of personal income in the data.

### Statistical analysis

Descriptive analysis was used to summarize primary care utilization and expenditures among multimorbid adults with different characteristics in reporting results as the frequency and percentage. Differences in categorical variables between groups were assessed by a chi-square test. A two-part model was applied to estimate determinants affecting the utilization and OOPE of primary care among multimorbid patients, by adjusted robust standard errors, odds ratios (OR), and coefficients of GLM. In addition, multiple imputation of Monte Carlo simulations was performed on the missing data to obtain the interpolated dataset, and each interpolated data set was analyzed to verify the robustness of the original model. Use the TPM command for data analysis and the margin command to obtain marginal effects in STATA 16.0 (Stata Corp.). The level of statistical significance was set as *P* = 0.05.

Previous studies have confirmed that medical expenditures are semi-continuous quantitative data, including zero and non-zero positive values with highly right-biased distribution (42, 43). In this case, the two-part model is more suitable than linear regression. Linear regression requires satisfying diverse regression assumptions, and its use may lead to biased estimates of the relationship between the outcome variables and observed variables. In contrast, two-part models are highly flexible and combine the advantages of parametric and non-parametric regressions (44), without the requirement to follow normal distribution and homoskedasticity criteria, facilitating the handling of highly complex medical costs and obtaining best-fit and unbiased estimations.

The two-part model divided the decision-making process into two steps. The first step applied a logit model to estimate the probability of using primary outpatient or inpatient services, and the second step adopted a GLM with gamma distribution and a logit link function to analyze the determinants of OOPE. Since the GLM directly provides estimates without any transformation (42). During data processing, 61 outpatients and 21 inpatients with zero medical expenditures, due to health insurance paid for all of the patients' medical expenses. The subsequent solution was to add a constant of 1 to the presence of true zeros so as to ensure that these samples entered the second stage of modeling (29, 45). The two-part model was as follows:


$$\begin{array}{l} P_{r}\left(c_{i}>0/x_{i}\right)=e^{\alpha +\beta X_{i}}/1+e^{\alpha +\beta X_{i}} \end{array}$$

*P*_*r*_ denotes individual healthcare decision-making behavior, *x*_*i*_ denotes sociodemographic and health factors influencing primary care utilization. *P*_*r*_ is a binary variable, if *c*_*i*_ > 0 means that patients seek treatment from primary care institutions, *P*_*r*_ = 1; if otherwise, it is *P*_*r*_ = 0.


$$\begin{array}{l} \text{GLM}\;\text{with}\;\text{a}\;\text{logit}\;\text{link}:c_{i}=e^{\alpha +\beta X_{i}+\varepsilon_{i}} \end{array}$$

*c*_*i*_ denotes the logarithm of primary outpatient and inpatient expenses; *x*_*i*_ denotes determinants of primary care utilization and expenses. ε_*i*_ is the random error term.

The final form of the two-part model is given by:


$$\begin{array}{l} E\left(c_{i}/x_{i}\right)=P_{r}\left(c_{i}>0/x_{i}\right)E\left(c_{i}/x_{i},c_{i}>0\right) \end{array}$$

### Patient and public involvement

The Biomedical Ethics Review Committee of Peking University approved CHARLS (IRB00001052-11015). No participants were involved in designing the study, analyzing the results, or writing the paper.

## Results

### Demographic characteristics

Table 1 presents the characteristics of the multimorbid elderly. A total of 4,384 elderly with multimorbidity, with the largest number of two chronic diseases (39.14%), followed by three chronic diseases (23.36%). Among the elderly, 41.54% were 70~79 years old, 51.05% were women, 77.74% were married and 74.11% were rural patients. In terms of education, 28.10% were illiterate, and 52.37% had primary education, implying that the vast majority of multimorbid patients had a low level of education. Concerning health insurance, 21.72% were uninsured, 9.58% had UEBMI, and 62.68% had URBMI. Nearly 90% reported that their health was fair or poor, 60.79% were satisfied with their health, and 49.5% exercised regularly.

**Table 1:** Characteristics among the multimorbid elderly.

**Variables**		***N***	**Percent (%)**
Age	60~69	1,680	38.32
	70~79	1,821	41.54
	80~	883	20.14
Gender	Man	2,146	48.95
	Woman	2,238	51.05
Education	Illiterate	1,232	28.10
	Primary school	2,296	52.37
	Middle school	337	7.69
	High school and above	519	11.84
Marital status	Unmarried	976	22.26
	Married	3,408	77.74
Residence	Central of city/town	815	18.59
	Urban–rural integration area	304	6.93
	Rural area	3,249	74.11
	Special area	16	0.36
Health insurance	No insurance	952	21.72
	UEBMI	420	9.58
	URBMI	2,748	62.68
	Two and above	195	1.57
	Other	69	4.45
Household economy	0~299	1,036	23.63
	299~657	1,049	23.93
	657~880	1,394	31.80
	880~1,510	905	20.64
Self-rated health	Very good	166	3.79
	Good	278	6.34
	Fair	1,929	44.00
	Poor	2,011	45.87
Health satisfaction	No	1,719	39.21
	Yes	2,665	60.79
Exercise	No	2,214	50.50
	Yes	2,170	49.50
Obs		4,384	100.00

### Utilization and OOPE of primary care

**Figure 2:**
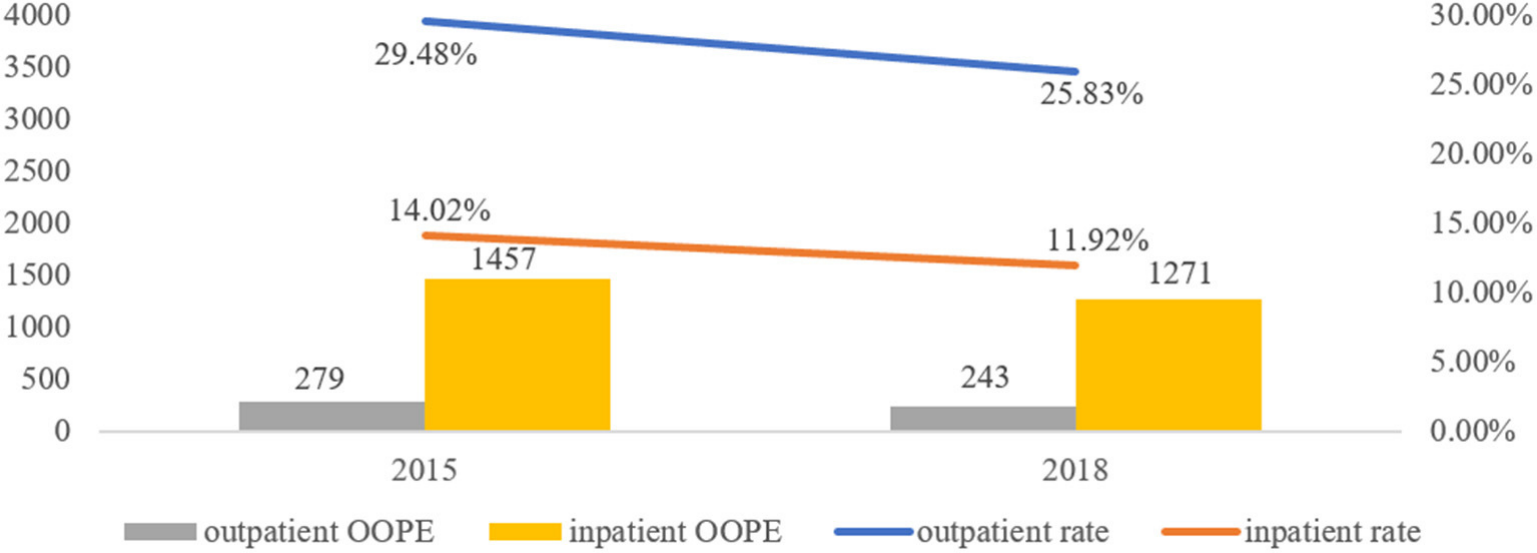
Primary care utilization and OOPE among the multimorbid elderly from 2015 to 2018.

Figure 2 demonstrates the changes of the visit rate and OOPE from 2015 to 2018. The primary outpatient visits rate of multimorbid patients decreased from 29.48% in 2015 to 25.83% in 2018, and the inpatient rate diminished from 14.02 to 11.92%. The outpatient OOPE reduced from 279 RMB in 2015 to 243 RMB in 2018, and inpatient OOPE dropped from 1,457 to 1,271 RMB.

Table 2 shows the sample characteristics regarding utilization and OOPE of primary outpatient care. There were 1,232 multimorbid elderly using primary outpatient care. Individuals who were 60~69 years old, female, less educated, married, covered by EUBMI, with poor self-rated health, and occasional exercises used more primary outpatient care. The chi-square test proved that there were significant differences in primary outpatient services utilization among all variables. The OOPE was higher for patients who were over 80 years old, male, better educated, married, living in urban areas, had URBMI or without insurance, better household economy, poor self-rated health, dissatisfied with health status, and occasional exercises. Further, the average OOPE differed greatly in certain variables. Patients aged over 80 years spent 1.5 times as much as those aged 70~79 years. Those with a high school and above education spent about twice as much as those were illiterate. Urban patients spent ~2.4 times more than rural patients.

**Table 2:** Characteristics of primary outpatient care utilization and OOPE among the multimorbid elderly (*N* = 1,232).

**Variables**	***N***	**Outpatient rate (%)**	***P*-value**	**Mean OOPE(RMB)**
Age			< 0.001	
60~69	518	30.83		241
70~79	515	28.28		219
80~	199	22.54		407
Gender			< 0.001	
Man	505	23.53		280
Woman	727	32.48		243
Education			< 0.001	
Illiterate	400	32.47		229
Primary school	669	29.14		282
Middle school	54	16.02		322
High school and above	109	21.00		447
Marital status			< 0.001	
Unmarried	202	20.70		240
Married	1,030	30.22		262
Residence			< 0.001	
Central of city/town	144	30.83		522
Urban–rural integration area	58	19.08		230
Rural area	1,027	31.61		221
Special area	3	18.75		825
Health insurance			< 0.001	
No insurance	215	22.58		267
UEBMI	88	20.95		213
URBMI	864	31.44		268
Two and above	41	21.03		205
Other	24	34.78		98
Household economy			< 0.001	
0~298	177	17.08		204
299~656	182	17.35		188
657~879	761	54.59		269
880~1,510	112	12.38		376
Self-rated health			< 0.001	
Very good	23	13.86		160
Good	54	19.42		173
Fair	490	25.40		183
Poor	665	33.07		324
Health satisfaction			< 0.001	
No	538	31.30		273
Yes	694	26.04		247
Exercise			< 0.001	
No	714	32.25		204
Yes	518	23.87		334

Table 3 illustrates the characteristics concerning use and OOPE of primary inpatient care. A total of 580 multimorbid elderly used primary inpatient care. Older adults who were 70~79 years old, less educated, married, living in rural areas, insured, better household economy, fair or poor self-rated health, and dissatisfied with their health used more primary inpatient services. The chi-square test showed that except for gender and age, other variables were greatly associated with the utilization of primary inpatient care. Patients who were 60~69 years old, less educated, uninsured, better household economy, and dissatisfied with their health occurred higher OOPE. Notably, the elderly with the best household economy spent ~1.6 times as much as those with the worst household economy.

**Table 3:** Characteristics of primary inpatient care utilization and OOPE among the multimorbid elderly (*N* = 580).

**Variables**	***N***	**Inpatient rate (%)**	***P*-value**	**Mean OOPE(RMB)**
Age			0.180	
60~69	212	12.62		1,437
70~79	261	14.33		1,256
80~	107	12.12		1,328
Gender			0.151	
Man	300	13.98		1,210
Woman	280	12.51		1,470
Education			< 0.001	
Illiterate	189	15.34		1,454
Primary school	317	13.81		1,331
Middle school	17	5.04		1,209
High school and above	57	10.98		1,004
Marital status			0.007	
Unmarried	104	10.66		1,327
Married	476	13.97		1,337
Residence			< 0.001	
Central of city/town	74	9.08		1,351
Urban–rural integration area	30	9.87		1,499
Rural area	475	14.62		1,319
Special area	1	6.25		3,001
Health insurance			< 0.001	
No insurance	67	7.04		1,660
UEBMI	74	17.62		1,354
URBMI	413	15.03		1,285
Two and above	14	7.18		1,300
Other	12	17.39		1,195
Household economy			< 0.001	
0~298	68	6.56		959
299~656	68	6.48		1,322
657~879	394	28.26		1,368
880~1,510	50	5.52		1,599
Self-rated health			< 0.001	
Very good	10	6.02		1,303
Good	16	5.76		1,857
Fair	200	10.37		1,261
Poor	354	17.60		1,355
Health satisfaction			< 0.001	
No	284	16.52		1,409
Yes	296	11.11		1,265
Exercise			< 0.001	
No	378	17.07		1,339
Yes	202	9.31		1,210

### Predictors of primary care utilization and OOPE

Table 4 shows the predictors of primary outpatient and OOPE. Older adults, especially those over 80 years old, were significantly less likely to use primary outpatient services (*OR* = 0.80, *P* < 0.05). It is of great significance that participants who were women (*OR* = 1.51, *P* < 0.001), married (*OR* = 1.24, *P* < 0.05) and living in rural areas (*OR* = 1.77, *P* < 0.001) were more likely to use primary outpatient care. And the OOPE was significantly lower for those who lived in urban-rural integration areas (β = −0.66, *P* < 0.05) or rural areas (β = −0.72, *P* < 0.05) compared to those who lived in rural areas. Older people with fair or poor self-rated health were 1.9 times or 2.23 times more likely to use primary outpatient care than those with very good self-rated health (*OR* = 1.90, *P* < 0.001; *OR* = 2.23, *P* < 0.001), and the former had significantly higher OOPE than the latter (β = 0.62, *P* < 0.001). In addition, patients with middle school education had a significantly lower propensity to use primary outpatient services and occurred lower OOPE compared to illiterate patients (*OR* = 0.61, *P* < 0.001; β = −0.47, *P* < 0.001). Besides, patients with better household economy were significantly less likely to use primary outpatient care (*OR* = 0.96, *P* < 0.001), but may incur higher OOPE (β = 0.29, *P* < 0.05; β = 0.58, *P* < 0.05).

**Table 4:** Predictors of primary outpatient care utilization and OOPE among the multimorbid elderly.

**Variables**	**Logit**		**Glm**		**Marginal effect**
	**OR**	**RE**	**Coef**.	**RE**	***dy*/*dx***
Age (ref 60~69)
70~79	0.92^**^	0.08	−0.14	0.10	−13^*^
80~	0.80^**^	0.09	0.11	0.18	−2
Gender (ref man)					
Woman	1.51^***^	0.12	0.04	0.01	20^***^
**Education (ref illiterate)**
Primary school	0.95	0.08	*0.11*	*0.11*	6
Middle school	0.61^***^	0.11	−0.47^**^	0.20	−38^***^
High school and above	0.78^*^	0.11	−0.15	0.18	−1
**Marital status (ref unmarried)**
Married	1.24^**^	0.13	−0.06	0.14	5
**Residence (ref central of city/town)**
Urban–rural integration area	0.98	0.19	−0.66^**^	0.33	−48
Rural area	1.77^***^	0.21	−0.72^**^	0.29	31
Special area	1.05	0.77	0.22	0.74	28
**Health insurance (ref no insurance)**
UEBMI	0.78	0.13	−0.38^*^	0.23	−30^**^
URBMI	1.17	0.11	−0.04	0.15	3
Two and above	0.91	0.29	−0.91	0.24	−29
Other	1.58^*^	1.10	−0.44^***^	0.32	−35^**^
**Household economy (ref 0** **~** **298)**
299~656	1.10	0.13	−0.01	0.16	3
657~879	0.96^***^	0.62	0.29^**^	0.15	105^***^
880~1,510	0.91	0.13	0.58^**^	0.26	21
**Self-rated health (ref very good)**
Good	1.64^*^	0.45	0.05	0.29	14
Fair	1.90^***^	0.44	0.14	0.20	22^***^
Poor	2.23^***^	0.54	0.62^***^	0.23	64^***^
**Health satisfaction (ref no)**
Yes	1.10	0.09	−0.09	0.11	−2
**Exercise (ref no)**
Yes	0.92	0.07	−0.30^***^	0.11	18
Constant	0.40^***^	0.13	5.75^***^	0.37	
Obs	4,384		1,232		
Prob > chi^2^	< 0.001				
Pseudo R^2^	16.17				

*** Significant at 1%;

** significant at 5%;

* significant at 10%.

Marginal effect of primary outpatient care manifested that the cost of patients aged 70~79 years was significantly less than those aged 60~69 years by 13 RMB. The expenditures for adults with middle school education were of remarkable significance lower than that of illiterate by 38 RMB. Patients covered by UEBMI or other insurance paid significantly less than those without health insurance by 30 and 35 RMB, respectively. The cost for women was of great significance higher than that for men by 20 RMB. It is significant that respondents with better household economy spent 105 RMB more than those with the worst household economy. Compared to those with good self-rated health, the expenses of respondents with fair or poor self-rated health were significantly higher by 22 and 64 RMB, respectively.

Table 5 revealed the predictors of primary inpatient care. Women were less likely than men to be hospitalized (*OR* = 0.74, *P* < 0.001), but may occur higher OOPE (β = 0.18, *P* < 0.05). Individuals with middle school education (*OR* = 0.40, *P* < 0.001) and better household economy (*OR* = 0.04, *P* < 0.001) had a significantly lower propensity to use primary inpatient care. Regarding cost, OOPE was significantly lower for those with high school or higher education (β = −0.41, *P* < 0.001) whereas higher OOPE may occur among those with better household economy (β = 0.40, *P* < 0.001; β = 0.62, *P* < 0.001). The likelihood of using primary inpatient care for those who had UEBMI (*OR* = 2.46, *P* < 0.001), URBMI (*OR* = 1.81, *P* < 0.001), and other insurance (*OR* = 2.15, *P* < 0.05) were 2.46, 1.81, and 2.15 times than the uninsured group. And OOPE was significantly lower in the URBMI group than in the uninsured group (β = −0.25, *P* < 0.05). It is significant that seniors who living in rural areas (*OR* = 1.48, *P* < 0.001), with poor self-rated health (*OR* = 2.30, *P* < 0.001) were more greatly likely to use primary inpatient care, and those who were satisfied with their health would have lower OOPE (β = −0.21, *P* < 0.05).

**Table 5:** Predictors of primary inpatient care utilization and OOPE among the multimorbid elderly.

**Variables**	**Logit**		**Glm**		**Marginal effect**
	**OR**	**RE**	**Coef**.	**RE**	***dy/dx***
**Age (ref 60** **~** **69)**
70~79	1.18	0.13	−0.16	0.10	−6
80~	1.13	0.09	–−0.03	0.12	11
Gender (ref man)					
Woman	0.74^***^	0.07	0.18^**^	0.09	−8
**Education (ref illiterate)**
Primary school	0.86	0.08	−0.05	0.10	−31
Middle school	0.40^***^	0.11	−0.27	0.17	−135^***^
High school and above	0.78	0.11	−0.41^***^	0.15	−84^***^
**Marital status (ref unmarried)**
Married	0.87	0.11	0.03	0.11	−14
**Residence (ref central of city/town)**
Urban–rural integration area	1.11	0.26	0.17	0.22	45
Rural area	1.48	0.22	−0.12	0.22	30^**^
Special area	0.81	0.95	0.86^***^	0.22	148
**Health insurance (ref no insurance)**
UEBMI	2.46^***^	0.48	−0.19^**^	0.18	95^**^
URBMI	1.81^***^	0.26	−0.25^**^	0.13	37
Two and above	0.93	0.30	−0.34^*^	0.24	44
Other	2.15^**^	0.84	−0.38	0.25	−52
**Household economy (ref 0** **~** **298)**
299~656	1.03	0.18	0.33^*^	0.17	26
657~879	0.04^***^	0.73	0.40^***^	0.23	29^***^
880~1,510	0.99	0.20	0.62^***^	0.12	50^**^
**Self-rated health (ref very good)**
Good	0.85	0.36	0.11	0.30	19
Fair	1.49	0.51	0.13	0.22	26
Poor	2.30^***^	0.80	0.29	0.22	88^**^
**Health satisfaction (ref no)**
Yes	0.94	0.10	−0.21^**^	0.10	−45^**^
**Exercise (ref no)**
Yes	0.69^***^	0.69	−0.04	0.09	−42^**^
Constant	0.04^**^	0.01	7.38^***^	0.32	
Obs	4,384		580		
Prob > chi^2^	< 0.001				
Pseudo R^2^	14.54				

*** Significant at 1%;

** significant at 5%;

* significant at 10%.

Marginal effects of primary inpatient care indicated that patients with middle school education or high school and above education spent significantly less than illiterate patients by 135 and 84 RMB, respectively. It is of great significance that participants who were satisfied with their health had lower expenditures of 45 RMB than those who were dissatisfied with their health. The cost for those who exercised regularly was significantly lower than for those who exercised occasionally by 42 RMB. It is of great significance that rural patients spent 30 RMB more than urban persons. Individuals with better or best household economy cost significantly more than those with the worst household economy by 29 and 50 RMB, respectively. It is significant that patients with poor self-rated health had higher spending of 88 RMB than those with very good self-rated health.

### Robustness test

In order to further verify the robustness of the above findings, this study performed multiple imputation on missing data and obtained some datasets, regressed each dataset separately and verified the robustness of the results. The results indicated that the relationship between the observed variables and utilization and cost is more consistent with the original conclusions, which strongly validated the reliability of the original outcomes. The regression results for the interpolated data also demonstrated that gender, education, household economy, and self-rated health were all associated with primary care utilization, while education, residence, household economy, self-rated health and so on were associated with OOPE of primary care (shown in Table 6).

**Table 6:** Robustness test.

**Variables**	**Outpatient**		**Inpatient**	
	**OR**	**Coef**.	**OR**	**Coef**.
**Age (ref 60** **~** **69)**
70~79	0.95^**^	−0.14	1.78	−0.23
80~	0.74^**^	0.22	2.57	−0.03
**Gender (ref man)**
Woman	2.58^***^	0.04	0.74^***^	0.28^**^
**Education (ref illiterate)**
Primary school	0.92	0.22	0.67	−0.05
Middle school	0.74^***^	−0.47^**^	0.73^***^	−0.27
High school and above	0.65^**^	−0.25	0.85^**^	−0.42^***^
Marital status (ref unmarried)
Married	2.37^**^	−0.06	0.95	0.03
**Residence (ref central of city/town)**
Urban–rural integration area	0.98^*^	−0.65^**^	2.46	0.27
Rural area	2.94^***^	−0.73^**^	2.73	−0.22
Special area	2.02	0.28	0.78	0.86^***^
**Health insurance (ref no insurance)**
UEBMI	0.72	−0.32^*^	2.78^***^	−0.29^**^
URBMI	2.25	−0.23	2.92^***^	−0.25^**^
Two and above	0.82	−0.84	1.23	−0.34^*^
Other	2.67^*^	−0.44^***^	2.34^**^	−0.38
**Household economy (ref 0** **~** **298)**
299~656	2.28	0.02	2.03	0.36^**^
657~879	0.94^***^	0.30^**^	0.04^***^	0.37^***^
880~2,520	0.92^**^	0.67^**^	0.99^**^	0.45^***^
**Self-rated health (ref very good)**
Good	2.45^**^	0.05	1.45	0.22
Fair	2.98^***^	0.24^**^	1.91^**^	0.23^*^
Poor	2.27^***^	0.64^***^	2.21^***^	0.29^**^
**Health satisfaction (ref no)**
Yes	2.35	−0.09	0.94	−0.22^**^
**Exercise (ref no)**
Yes	0.98	−0.30^***^	0.69^***^	−0.12
Constant	0.40^***^	5.75^***^	0.04^**^	7.38^***^
Obs	6,065		6,065	
Prob > chi^2^	< 0.001		< 0.001	
Pseudo R^2^	13.37		12.35	

*** Significant at 1%;

** significant at 5%;

* significant at 10%.

## Discussion

Globally, health systems are increasingly challenged to care for older adults with complex multimorbidity. The study used nationally representative data and a two-part model to clarify current primary care utilization, OOPE and its associated impacts on the multimorbid elderly. The primary outpatient visits rate of multimorbid patients dropped from 29.48% in 2015 to 25.83% in 2018, and the inpatient rate reduced from 14.02 to 11.92%. The outpatient OOPE declined from 279 RMB in 2015 to 243 RMB in 2018, and inpatient OOPE decreased from 1,451 to 1,271 RMB. On the one hand, primary outpatient and inpatient utilization were both influenced by gender, marital status, education, self-rated health and household economy, whereas health insurance and exercise frequency also were related to inpatient utilization. On the other hand, education, residence, household economy, self-rated health and exercise frequency were associated with primary outpatient expenditures, as well as gender, education, health insurance, household economy and health satisfaction were relevant to primary inpatient expenses.

In this study, primary care visit rates and OOPE trended downward from 2015 to 2018. Yang et al. (46) observed a decreasing trend in primary outpatient visits whereas an increasing trend in large hospital outpatient visits among the middle-aged and older adults in Chinese referral system. Ganguli et al. (47) surveyed primary care utilization among commercially insured adults and found a 24.2% decrease in primary care visits and a $9.4 increase in OOPE per visit. But there are some studies confirmed that a sound primary care service system is conducive to the reduction of OOPE (48, 49). It is well-known that the primary care improves health outcomes and reduces economic burden. Despite a series of favorable policies to launch a hierarchical medical system in China, the dearth of decentralization of professionals and medical technology to the primary healthcare facilities (50), and the lack of change in perceptions of primary care visits have led to a decline in the utilization of primary care services. Possible explanations for the shrinkage in OOPE are that the continuity treatment in primary care institutions prevents the escalation of disease severity among multimorbid patients and higher health insurance reimbursement in primary care institutions than in secondary and tertiary hospitals (51), thereby reducing the financial burden on patients.

### Factors related to primary outpatient care utilization and OOPE

This study reported that older adults aged 80 and above used fewer primary outpatient services. Due to biological aging, decline in physiological function, and exposure and accumulation of risk factors throughout the lifespan, the elderly place higher expectations on healthcare (52). Furthermore, this study also reported that patients who are female, married, and living in rural areas tended to utilize more primary outpatient care, which was in line with the result of a Swedish study that females more frequently use primary care (53). The plausible reasons may be that women have strong perceptions of illness for their physical vulnerability (28), and the convenient accessibility of primary care facilities stimulates them to use primary outpatient care. Thirdly, as demonstrated in this study, married populations were more likely to use primary outpatient care, which was supported by a longitudinal study that assessed the relationship between marriage and multimorbidity across countries (54). The benefit of marriage is that spouses may provide adequate financial and psychological support, and promptly urge and accompany them to receive the necessary treatment (55). Fourthly, consistent with a prior study (56), the result suggested that rural residents were more likely to use primary outpatient services. In most cases, patients who lived in rural areas are susceptible to transportation restrictions, and are trust in primary physicians with fewer communication barriers such as professionalism and terminology understanding (57), which increase primary outpatient care visits by rural patients. Finally, the results from this study suggested that the impact of education and household economy were negative for frequent primary outpatient utilization. Since the healthcare system in China allows patients freely to choose any healthcare institution (58), patients with higher education and better household economy are more likely to choose large hospitals where they can provide better care.

The research assumed that rural patients had lower OOPE than urban patients. Compared to urban areas, rural primary care facilities are mostly equipped with drugs from the national essential drug list and have relatively more low-priced drugs (12), so the cost may be reduced. Additionally, as reported in this study, since more educated patients with better health literacy are able to effectively reduce the risk of multimorbidity and improve physical function by managing health risk factors, education was negatively associated with OOPE. And some prior studies indicated that household economy is one of the powerful factors affecting expenses (59, 60). Wealthy households have more disposable funds and are less constrained by medical prices during treatment, which in turn consumes more and more expensive drugs or tests resulting in higher OOPE. Besides, this study also demonstrated that patients who perceived themselves to be in better health and exercised regularly had lower OOPE. The plausible reason may be that the ones who have positive attitudes toward their health, and proactively regulate their lifestyle and mindset, tends to achieve protective health outcomes by reducing functional impairment through moderate health activities (61), which subsequently reduce healthcare costs.

### Factors related to primary inpatient care utilization and OOPE

This study confirmed the results of previous research that women were significantly less likely to use inpatient services than men (62). The plausible reason may be that many women in China are fully engaged with families and do not have sufficient income to afford the relatively expensive inpatient expenses (63). Besides, similar to previous studies (59, 64), those with higher education and better household economy were negatively correlated with primary inpatient care utilization. Although some other prior research indicated a positive association between income and healthcare utilization in LMICs (65), this pattern is more applicable to secondary and tertiary hospitals than to primary hospitals. This is because the purchasing power of patients with better education and higher incomes increase their access to healthcare in secondary or tertiary hospitals. Additionally, this study also demonstrated that different types of health insurance had different effects on primary inpatient care utilization, which was consistent with previous findings (30, 66). Patients with health insurance generally had a higher utilization than those uncovered by any health insurance, because health insurance improves their affordability by paying a proportion of total health expenditure (67, 68). Similar to existing empirical study (66), those with health insurance were more likely to utilize primary inpatient care while health insurance was insignificant in driving primary outpatient use. The rational explanation is that outpatient claims cover fewer types of illness and lower reimbursement rates for outpatient care (28). Finally, in conformity with prior studies (69, 70), individuals who lacked exercise and had poorer self-rated health used healthcare more frequently than those who exercise in moderation. A reasonable explanation is that inadequate exercise or poor self-rated health can directly affect health outcomes in older adults, leading to more primary hospitalization utilization.

Similar to the results of previous studies (62, 71), women have significantly higher OOPE than men. Compared with men, women have a higher probability to delay hospitalization for family or financial reasons, increasing the severity of diseases and causing more complications, which result in an escalation of expenditures. Moreover, this study also found that OOPE was higher for those with better household economy. This is because patients with better household economy place more value on the quality of medical care provided by medical institutions and post-treatment health outcomes than on medical expenses (72, 73). In accordance with previous research (74), the study also proved that the OOPE of patients with health insurance was significantly lower than those uncovered by any health insurance. The reason is that a proportion of medical expenses can be reimbursed by health insurance, which will directly reduce the economic burden related to hospitalization. Besides, patients with UEBMI had higher OOPE than those with URBMI in this study. Empirical studies in China (75), Germany (76) and Thailand (77) also reported a similar situation, that is, medical insurance with more generous reimbursement is prone to occur higher OOPE. And UEBMI targets groups with relatively higher social status and easier access to treatment. In addition, the finding of this study suggested that individuals who were more satisfied with their health status could better control medical expenses, as positive attitudes occur more health-promoting behaviors and decrease expenses (78).

### Policy implications

By 2050, the elderly population in China will account for half of the total population (79), which may result in an epidemic of chronic diseases in the elderly. Thus, the country needs to adjust its disease management strategies in advance to prevent the prevalence of complex multimorbidity. Then, focus on vulnerable groups such as women, less educated and poorer self-rated health, and take targeted interventions to enhance their awareness of disease prevention and reduce inappropriate medical expenses. Since the geographical constraints and urban-rural disparities would exacerbate inequality in medical services and expenses, consequently local governments or other social organizations need to provide additional financial, professional and equipment support to bridge the gap in the quality of health services between rural and urban areas. Finally, given the prevalence of multimorbidity and healthcare systems still oriented toward a single disease, patient-centered care should be reconsidered and emphasized to more effectively manage comorbidities and improve the quality of life (80, 81).

### Strengths and limitations

The strengths of this study were as follows. First, since few studies has emphasized and segmented primary outpatient and inpatient service utilization and expenditure among elderly patients with multimorbidity, focusing on the multimorbid groups with severe disease burden can fill the corresponding research gap. And it will also benefit strengthening of primary management of chronic diseases and advancing the construction of an orderly hierarchical medical system. Second, guided by the health demand model of Grossman theory to screen variables, this study ensures that the variable selection takes into account the influence of health demand on subsequent behavior and the scientific factor inclusion, which will overcome the shortcoming of ignoring the impact of health demand. Finally, a two-part model was introduced to investigate the determinants of primary care utilization and expenses so as to systematically capture the current utilization status and economic burden among the multimorbid elderly. Two-part models have the flexibility to deal with the highly skewed and some zeros of health expenses to compensate for the shortcomings of single method such as linear regression (82, 83).

However, this study also had some limitations. First, the cross-sectional studies have their limitations in drawing causality conclusions. Future research will be recommended to implement data collection at different time points to improve causal inference. Furthermore, some variables in CHARLS were subjective judgments of participants, such as self-rated health and health satisfaction, hence the recall bias and the effect of social desirability can't be ruled out.

## Conclusion

Guided by Grossman theory, this study applied a two-part model to validate the determinants affecting primary care utilization and expenditures among the multimorbid elderly. The primary care visit rates and OOPE declined from 2015 to 2018. Determinants, such as gender, education, residence, health insurance, household economy and self-rated health were strongly associated with primary care utilization and expenditures. Other factors, such as health satisfaction, exercise frequency had fewer impacts on these. The findings will provide new evidence for developing targeted policies and interventions to promote rational utilization of primary care and reduce avoidable economic burdens.

## Data availability statement

Publicly available datasets were analyzed in this study. This data can be found here: https://charls.charlsdata.com/index/zh-cn.html.

## Author contributions

WL guided the whole process and reviewed the manuscript. YC carried out the data analysis and drafted the manuscript. All authors read and approved the manuscript before submission.

## Funding

This study is supported by the Natural Science Foundation of Fujian Province (Grant No. 2021J01245).

## Conflict of interest

The authors declare that the research was conducted in the absence of any commercial or financial relationships that could be construed as a potential conflict of interest.

## Publisher's note

All claims expressed in this article are solely those of the authors and do not necessarily represent those of their affiliated organizations, or those of the publisher, the editors and the reviewers. Any product that may be evaluated in this article, or claim that may be made by its manufacturer, is not guaranteed or endorsed by the publisher.

## References

[1] Zhao Y Zhao S Zhang L Haregu TN Wang H. Impacts of multimorbidity on medication treatment, primary healthcare and hospitalization among middle-aged and older adults in China: evidence from a nationwide longitudinal study. BMC Public Health. (2021) 21:1380. 10.1186/s12889-021-11456-7 34253222 PMC8274017

[2] Feinstein AR. The pre-therapeutic classification of co-morbidity in chronic disease. J Chronic Dis. (1970) 23:455–68. 10.1016/0021-9681(70)90054-8 26309916

[3] Abebe F Schneider M Asrat B Ambaw F. Multimorbidity of chronic non-communicable diseases in low- and middle-income countries: a scoping review. J Comorb. (2020) 10:2235042X20961919. 10.1177/2235042X20961919 33117722 PMC7573723

[4] Violan C Foguet-Boreu Q Flores-Mateo G Salisbury C Blom J Freitag M. Prevalence, determinants and patterns of multimorbidity in primary care: a systematic review of observational studies. PLoS ONE. (2014) 9:e102149. 10.1371/journal.pone.0102149 25048354 PMC4105594

[5] Xu X Mishra GD Jones M. Mapping the global research landscape and knowledge gaps on multimorbidity: a bibliometric study. J Glob Health. (2017) 7:010414. 10.7189/jogh.07.010414 28685036 PMC5475311

[6] Zhao YW Haregu TN He L Lu S Katar A Wang H. The effect of multimorbidity on functional limitations and depression amongst middle-aged and older population in China: a nationwide longitudinal study. Age Ageing. (2021) 50:190–7. 10.1093/ageing/afaa117 32556149

[7] Anindya K Ng N Atun R Marthias T Zhao Y McPake B. Effect of multimorbidity on utilisation and out-of-pocket expenditure in Indonesia: quantile regression analysis. BMC Health Serv Res. (2021) 21:427. 10.1186/s12913-021-06446-9 33952273 PMC8097787

[8] Ciminata G Geue C Langhorne P Wu O. A two-part model to estimate inpatient, outpatient, prescribing and care home costs associated with atrial fibrillation in Scotland. BMJ Open. (2020) 10:e028575. 10.1136/bmjopen-2018-028575 32193256 PMC7150597

[9] Aramrat C Choksomngam Y Jiraporncharoen W Wiwatkunupakarn N Pinyopornpanish K Mallinson PAC. Advancing multimorbidity management in primary care: a narrative review. Prim Health Care Res Dev. (2022) 23:e36. 10.1017/S1463423622000238 35775363 PMC9309754

[10] Kato D Ryu H Matsumoto T Abe K Kaneko M Ko M. Building primary care in Japan: literature review. J Gen Fam Med. (2019) 20:170–9. 10.1002/jgf2.252 31516802 PMC6732569

[11] Pollock AM Price D Viebrock E Miller E Watt G. The market in primary care. BMJ. (2007) 335:475–7. 10.1136/bmj.39303.425359.AD 17823186 PMC1971206

[12] Li C Chen Z Khan MM. Bypassing primary care facilities: health-seeking behavior of middle age and older adults in China. BMC Health Serv Res. (2021) 21:895. 10.1186/s12913-021-06908-0 34461884 PMC8406824

[13] Sanders SR Erickson LD Call VR McKnight ML Hedges DW. Rural health care bypass behavior: how community and spatial characteristics affect primary health care selection. J Rural Health. (2015) 31:146–56. 10.1111/jrh.12093 25219792

[14] Sang H Gonzalez-Vallejo C Zhao J Long R. Is low cost really conducive to primary care utilisation: an empirical analysis of community health centers in China. Health Soc Care Commun. (2021) 29:e163–73. 10.1111/hsc.13262 33386777

[15] Dai H Tang L Wang Z Sun X Zhang F Zhu M. Facilitate signing with the family doctor: a study of the practice in Shanghai, China. Int J Gen Med. (2021) 14:6907–17. 10.2147/IJGM.S332890 34703295 PMC8536883

[16] Guinness L Paul RC Martins JS Asante A Price JA Hayen A. Determinants of health care utilisation: the case of timor-leste. Int Health. (2018) 10:412–20. 10.1093/inthealth/ihy044 30007293 PMC6204763

[17] van den Bussche H Schon G Kolonko T Hansen H Wegscheider K Glaeske G. Patterns of ambulatory medical care utilization in elderly patients with special reference to chronic diseases and multimorbidity: results from a claims data based observational study in Germany. BMC Geriatr. (2011) 11:54. 10.1186/1471-2318-11-54 21914191 PMC3180370

[18] Ahn S Bartmess M Lindley LC. Multimorbidity and healthcare utilization among black Americans: a cross-sectional study. Nurs Open. (2022) 9:959–65. 10.1002/nop2.1095 34935300 PMC8859074

[19] Acharya S Ghimire S Jeffers EM Shrestha N. Health care utilization and health care expenditure of Nepali Older adults. Front Public Health. (2019) 7:24. 10.3389/fpubh.2019.00024 30828573 PMC6384236

[20] Lueckmann SL Hoebel J Roick J Markert J Spallek J von dem Knesebeck O. Socioeconomic inequalities in primary-care and specialist physician visits: a systematic review. Int J Equity Health. (2021) 20:58. 10.1186/s12939-020-01375-1 33568126 PMC7874661

[21] Tille F Gibis B Balke K Kuhlmey A Schnitzer S. [Sociodemographic and health-related determinants of health care utilisation and access to primary and specialist care: results of a nationwide population survey in Germany (2006–2016)]. Z Evid Fortbild Qual Gesundhwes. (2017) 126:52–65. 10.1016/j.zefq.2017.07.012 28916160

[22] Li C Zhou R Yao N Cornwell T Wang S. Health care utilization and unmet needs in Chinese older adults with multimorbidity and functional impairment. J Am Med Dir Assoc. (2020) 21:806–10. 10.1016/j.jamda.2020.02.010 32222351

[23] Gotsadze G Zoidze A Rukhadze N Shengelia N Chkhaidze N. An impact evaluation of medical insurance for poor in Georgia: preliminary results and policy implications. Health Policy Plan. (2015) 30:i2–13. 10.1093/heapol/czu095 25759451

[24] Davis-Ajami ML Lu ZK Wu J. Multiple chronic conditions and associated health care expenses in us adults with cancer: a 2010–2015 medical expenditure panel survey study. BMC Health Serv Res. (2019) 19:981. 10.1186/s12913-019-4827-1 31856797 PMC6924021

[25] Breyer F Lorenz N. The “Red Herring” after 20 years: ageing and health care expenditures. Eur J Health Econ. (2021) 22:661–7. 10.1007/s10198-020-01203-x 32500244 PMC8214577

[26] Seshamani M Gray A. Ageing and health-care expenditure: the red herring argument revisited. Health Econ. (2004) 13:303–14. 10.1002/hec.826 15067669

[27] Lee JT Hamid F Pati S Atun R Millett C. Impact of noncommunicable disease multimorbidity on healthcare utilisation and out-of-pocket expenditures in middle-income countries: cross sectional analysis. PLoS ONE. (2015) 10:e0127199. 10.1371/journal.pone.0127199 26154083 PMC4496037

[28] Wang Z Li X Chen M Si L. Social health insurance, healthcare utilization, and costs in middle-aged and elderly community-dwelling adults in China. Int J Equity Health. (2018) 17:17. 10.1186/s12939-018-0733-0 29394933 PMC5797397

[29] Caballer-Tarazona V Guadalajara-Olmeda N Vivas-Consuelo D. Predicting healthcare expenditure by multimorbidity groups. Health Policy. (2019) 123:427–34. 10.1016/j.healthpol.2019.02.002 30791988

[30] Fan G Deng Z Wu X Wang Y. Medical insurance and health equity in health service utilization among the middle-aged and older adults in China: a quantile regression approach. BMC Health Serv Res. (2020) 20:553. 10.1186/s12913-020-05423-y 32552901 PMC7302153

[31] Zhou XH Liang H. Semi-parametric single-index two-part regression models. Comput Stat Data Anal. (2006) 50:1378–90. 10.1016/j.csda.2004.12.001 20191094 PMC2828618

[32] Grossman M. On the concept of health capital and the demand for health. J Polit Econ. (1972) 80:223–55. 10.1086/259880

[33] Masiye F Kaonga O. Determinants of healthcare utilisation and out-of-pocket payments in the context of free public primary healthcare in Zambia. Int J Health Policy Manag. (2016) 5:693–703. 10.15171/ijhpm.2016.65 28005549 PMC5144876

[34] Grossman M. The demand for health turns 50: reflections. Health Econ. (2022) 31:1807–22. 10.1002/hec.4563 35801541

[35] Bala MM Singh S Kumar N Janor H. Predicting key drivers for health care expenditure growth in the middle east region: a grossman-pls modeling approach. Exp Rev Pharmacoecon Outcomes Res. (2022) 22:1021–31. 10.1080/14737167.2022.2073222 35491846

[36] Hartwig J Sturm JE. Testing the grossman model of medical spending determinants with macroeconomic panel data. Eur J Health Econ. (2018) 19:1067–86. 10.1007/s10198-018-0958-2 29453763

[37] Morera Salas M Aparicio Llanos A. [Determinants of health care utilization in costa rica]. Gac Sanit. (2010) 24:410–5. 10.1016/j.gaceta.2010.05.009 20934785

[38] Galama TJ van Kippersluis H. Health inequalities through the lens of health-capital theory: issues, solutions, and future directions. Health Inequality. (2013) 21:263–84. 10.1108/S1049-2585(2013)0000021013 24570580 PMC3932058

[39] Sorkin AL. Some economic aspects of the demand for health services. Gaoxiong yi xue ke xue za zhi Kaohsiung J Med Sci. (1989) 5:610–20. 2699334

[40] Li C Tang C Wang H. Effects of health insurance integration on health care utilization and its equity among the mid-aged and elderly: evidence from China. Int J Equity Health. (2019) 18:166. 10.1186/s12939-019-1068-1 31665019 PMC6820904

[41] Islam A Smyth R. Do fertility control policies affect health in old age? Evidence from China's one-child experiment. Health Econ. (2015) 24:601–16. 10.1002/hec.3047 24692342

[42] Neelon B O'Malley AJ Smith VA. Modeling zero-modified count and semicontinuous data in health services research part 1: background and overview. Stat Med. (2016) 35:5070–93. 10.1002/sim.7050 27500945

[43] Zhang B Liu W Zhang N Ash AS Allison JJ. A collection of marginalized two-part random-effects models for analyzing medical expenditure panel data: impact of the new cooperative medical scheme on healthcare expenditures in China. Stat Methods Med Res. (2019) 28:2494–523. 10.1177/0962280218784725 29945495

[44] Sauzet O Razum O Widera T Brzoska P. Two-part models and quantile regression for the analysis of survey data with a spike the example of satisfaction with health care. Front Public Health. (2019) 7:146. 10.3389/fpubh.2019.00146 31245346 PMC6579824

[45] Liu L Strawderman RL Cowen ME Shih YC A. flexible two-part random effects model for correlated medical costs. J Health Econ. (2010) 29:110–23. 10.1016/j.jhealeco.2009.11.010 20015560 PMC2824028

[46] Yang S Zhou M Liao J Ding X Hu N Kuang L. Association between primary care utilization and emergency room or hospital inpatient services utilization among the middle-aged and elderly in a self-referral system: evidence from the China health and retirement longitudinal study 2011–2018. Int J Environ Res Public Health. (2022) 19:2979. 10.3390/ijerph191912979 36232279 PMC9564952

[47] Ganguli I Shi Z Orav EJ Rao A Ray KN Mehrotra A. Declining use of primary care among commercially insured adults in the United States, 2008–2016. Ann Intern Med. (2020) 172:240–7. 10.7326/M19-1834 32016285

[48] Zhou M Liao J Hu N Kuang L. Association between primary healthcare and medical expenditures in a context of hospital-oriented healthcare system in China: a national panel dataset, 2012–2016. Int J Environ Res Public Health. (2020) 17:6917. 10.3390/ijerph17186917 32971840 PMC7558376

[49] Dusheiko M Gravelle H Martin S Rice N Smith PC. Does better disease management in primary care reduce hospital costs? Evidence from english primary care. J Health Econ. (2011) 30:919–32. 10.1016/j.jhealeco.2011.08.001 21893358

[50] Li X Lu J Hu S Cheng KK De Maeseneer J Meng Q. The primary health-care system in China. Lancet. (2017) 390:2584–94. 10.1016/S0140-6736(17)33109-4 29231837

[51] Zhang A Nikoloski Z Mossialos E. Does health insurance reduce out-of-pocket expenditure? Heterogeneity among China's middle-aged and elderly. Soc Sci Med. (2017) 190:11–9. 10.1016/j.socscimed.2017.08.005 28823943

[52] Balakrishnan S Karmacharya I Ghimire S Mistry SK Singh DR Yadav OP. Prevalence of multimorbidity and its correlates among older adults in eastern Nepal. BMC Geriatr. (2022) 22:425. 10.1186/s12877-022-03115-2 35570271 PMC9109315

[53] Ranstad K Midlov P Halling A. Importance of healthcare utilization and multimorbidity level in choosing a primary care provider in Sweden. Scand J Prim Health Care. (2014) 32:99–105. 10.3109/02813432.2014.929819 24939741 PMC4075024

[54] Wang D Li D Mishra SR Lim C Dai X Chen S. Association between marital relationship and multimorbidity in middle-aged adults: a longitudinal study across the US, UK, Europe, and China. Maturitas. (2022) 155:32–9. 10.1016/j.maturitas.2021.09.011 34876247

[55] Rendall MS Weden MM Favreault MM Waldron H. The protective effect of marriage for survival: a review and update. Demography. (2011) 48:481–506. 10.1007/s13524-011-0032-5 21526396

[56] Yip WC Wang H Liu Y. Determinants of patient choice of medical provider: a case study in Rural China. Health Policy Plan. (1998) 13:311–22. 10.1093/heapol/13.3.311 10187600

[57] Murray B McCrone S. An integrative review of promoting trust in the patient-primary care provider relationship. J Adv Nurs. (2015) 71:3–23. 10.1111/jan.12502 25113235

[58] Wu D Lam TP Lam KF Zhou XD Sun KS. Public views towards community health and hospital-based outpatient services and their utilisation in Zhejiang, China: a mixed methods study. BMJ Open. (2017) 7:e017611. 10.1136/bmjopen-2017-017611 29101139 PMC5695298

[59] Hone T Stokes J Trajman A Saraceni V Coeli CM Rasella D. Racial and socioeconomic disparities in multimorbidity and associated healthcare utilisation and outcomes in Brazil: a cross-sectional analysis of three million individuals. BMC Public Health. (2021) 21:1287. 10.1186/s12889-021-11328-0 34210313 PMC8252284

[60] Kabir MR. Adopting Andersen's behavior model to identify factors influencing maternal healthcare service utilization in Bangladesh. PLoS ONE. (2021) 16:e0260502. 10.1371/journal.pone.0260502 34843566 PMC8629289

[61] Song X Wu J Yu C Dong W Lv J Guo Y. Association between multiple comorbidities and self-rated health status in middle-aged and elderly Chinese: the China kadoorie biobank study. BMC Public Health. (2018) 18:744. 10.1186/s12889-018-5632-1 29907132 PMC6003165

[62] Peoples N Gong E Gautam K Khanal SN Kohrt BA Koirala S. Perception and use of primary healthcare services among people with cardiometabolic diseases in two resource-limited areas in Nepal: a mixed methods study. Front Public Health. (2021) 9:698030. 10.3389/fpubh.2021.698030 34631643 PMC8494788

[63] Kyomuhendo GB. Low use of rural maternity services in Uganda: impact of women's status, traditional beliefs and limited resources. Reprod Health Matters. (2003) 11:16–26. 10.1016/S0968-8080(03)02176-1 12800700

[64] Pati S Swain S Hussain MA van den Akker M Metsemakers J Knottnerus JA. Prevalence and outcomes of multimorbidity in South Asia: a systematic review. BMJ Open. (2015) 5:e007235. 10.1136/bmjopen-2014-007235 26446164 PMC4606435

[65] Makinen M Waters H Rauch M Almagambetova N Bitran R Gilson L. Inequalities in health care use and expenditures: empirical data from eight developing countries and countries in transition. Bull World Health Organ. (2000) 78:55–65. 10686733 PMC2560608

[66] Ren J Ding D Wu Q Liu C Hao Y Cui Y. Financial affordability, health insurance, and use of health care services by the elderly: findings from the China health and retirement longitudinal study. Asia Pac J Public Health. (2019) 31:510–21. 10.1177/1010539519877054 31610715

[67] Wang J Zhu H Liu H Wu K Zhang X Zhao M. Can the reform of integrating health insurance reduce inequity in catastrophic health expenditure? Evidence from China. Int J Equity Health. (2020) 19:49. 10.1186/s12939-020-1145-5 32245473 PMC7126184

[68] Dong W Zwi AB Bai R Shen C Gao J. Benefit of China's social health insurance schemes: trend analysis and associated factors since health reform. Int J Environ Res Public Health. (2021) 18:5672. 10.3390/ijerph18115672 34070687 PMC8199469

[69] Denkinger MD Lukas A Herbolsheimer F Peter R Nikolaus T. Physical activity and other health-related factors predict health care utilisation in older adults: the actife ulm study. Z Gerontol Geriatr. (2012) 45:290–7. 10.1007/s00391-012-0335-1 22622677

[70] Rocca P Beckman A Ekvall Hansson E Ohlsson H. Is the association between physical activity and healthcare utilization affected by self-rated health and socio-economic factors? BMC Public Health. (2015) 15:737. 10.1186/s12889-015-2079-5 26231379 PMC4522137

[71] Barrio-Cortes J Soria-Ruiz-Ogarrio M Martinez-Cuevas M Castano-Reguillo A Bandeira-de Oliveira M Beca-Martinez MT. Use of primary and hospital care health services by chronic patients according to risk level by adjusted morbidity groups. BMC Health Serv Res. (2021) 21:1046. 10.1186/s12913-021-07020-z 34600525 PMC8487403

[72] Adler NE Boyce T Chesney MA Cohen S Folkman S Kahn RL. Socioeconomic status and health the challenge of the gradient. Am Psychol. (1994) 49:15–24. 10.1037/0003-066X.49.1.15 8122813

[73] Mosadeghrad AM. Factors influencing healthcare service quality. Int J Health Policy Manag. (2014) 3:77–89. 10.15171/ijhpm.2014.65 25114946 PMC4122083

[74] Zhou Y Wushouer H Vuillermin D Ni B Guan X Shi L. Medical insurance and healthcare utilization among the middle-aged and elderly in China: evidence from the China health and retirement longitudinal study 2011, 2013, and 2015. BMC Health Serv Res. (2020) 20:654. 10.1186/s12913-020-05522-w 32664947 PMC7362522

[75] Chen S Lin Z Fan X Li J Xie YJ Hao C. The comparison of various types of health insurance in the healthcare utilization, costs and catastrophic health expenditures among middle-aged and older Chinese adults. Int J Environ Res Public Health. (2022) 19:5956. 10.3390/ijerph19105956 35627490 PMC9141905

[76] Krobot KJ Miller WC Kaufman JS Christensen DB Preisser JS Ibrahim MA. The disparity in access to new medication by type of health insurance: lessons from Germany. Med Care. (2004) 42:487–91. 10.1097/01.mlr.0000124265.13559.02 15083110

[77] Ngorsuraches S Ungsupanit J. Hpi the relationship between health insurance type and costs of prescribed drugs. Value Health. (2004) 7:651. 10.1016/S1098-3015(10)65678-1

[78] Mahmood A Kedia S Wyant DK Ahn S Bhuyan SS. Use of mobile health applications for health-promoting behavior among individuals with chronic medical conditions. Dig Health. (2019) 5:2055207619882181. 10.1177/2055207619882181 31656632 PMC6791047

[79] Guo AJ Ding XJ Zhong FL Cheng QP Huang CL. Predicting the future Chinese population using shared socioeconomic pathways, the sixth national population census, and a Pde model. Sustainability. (2019) 11:3686. 10.3390/su11133686

[80] Smith SM Wallace E O'Dowd T Fortin M. Interventions for improving outcomes in patients with multimorbidity in primary care and community settings. Cochrane Database Syst Rev. (2021) 1:CD006560. 10.1002/14651858.CD006560.pub4 33448337 PMC8092473

[81] Guthrie B Payne K Alderson P McMurdo ME Mercer SW. Adapting clinical guidelines to take account of multimorbidity. BMJ. (2012) 345:e6341. 10.1136/bmj.e6341 23036829

[82] Duan N Manning WG Morris CN Newhouse JP. Choosing between the sample-selection model and the multi-part model. J Bus Econ Stat. (1984) 2:283–9. 10.1080/07350015.1984.10509396

[83] Farewell VT Long DL Tom BDM Yiu S Su L. Two-part and related regression models for longitudinal data. Annu Rev Stat Appl. (2017) 4:283–315. 10.1146/annurev-statistics-060116-054131 28890906 PMC5590716

